# A double-layer hydrogel based on alginate-carboxymethyl cellulose and synthetic polymer as sustained drug delivery system

**DOI:** 10.1038/s41598-021-88503-1

**Published:** 2021-04-28

**Authors:** Yan Hu, Sheng Hu, Shangwen Zhang, Siyi Dong, Jie Hu, Li Kang, Xinzhou Yang

**Affiliations:** 1 School of Pharmaceutical Science, South-Central University for Nationalities, Wuhan, 430074 China; 2 National Demonstration Center for Experimental Ethnopharmacology Education, South-Central University for Nationalities, Wuhan, 430074 China

**Keywords:** Drug discovery, Materials science

## Abstract

A new double-layer, pH-sensitive, composite hydrogel sustained-release system based on polysaccharides and synthetic polymers with combined functions of different inner/outer hydrogels was prepared. The polysaccharides inner core based on sodium alginate (SA) and carboxymethyl cellulose (CMC), was formed by physical crosslinking with pH-sensitive property. The synthetic polymer out-layer with enhanced stability was introduced by chemical crosslinking to eliminate the expansion of inner core and the diffusion of inner content. The physicochemical structure of the double-layer hydrogels was characterized. The drug-release results demonstrated that the sustained-release effect of the hydrogels for different model drugs could be regulated by changing the composition or thickness of the hydrogel layer. The significant sustained-release effect for BSA and indomethacin indicated that the bilayer hydrogel can be developed into a novel sustained delivery system for bioactive substance or drugs with potential applications in drugs and functional foods.

A hydrogel is a three-dimensional network based on polymers. It has been applied in various fields, such as drug delivery^1^, tissue engineering^2^, cell engineering^3^, shape memory^4^, sensors^5^, and soft robots^6^. Stimuli-responsive hydrogels can control drug release owing to their chemical modification and adjustable network pore size^7,8^. Therefore, it is the most promising drug delivery platform. Oral administration remains the most tolerable route of drug administration for patients, but many drugs are unstable owing to the effects of pH and enzyme in the gastrointestinal tract^9^. As an oral drug delivery system, intelligent hydrogels can be used to protect drug activity and improve its bioavailability^10,11^. These intelligent materials can also become a trend in drug delivery in the future.

Compared with the traditional monolayer hydrogels, multilayer hydrogels have many advantages, such as multilayer structure, relatively independent layers, and controllable layers. It can better highlight its advantages to be developed as a drug carrier system^12,13^. In many studies, various materials have been used to prepare multilayer hydrogels, but these studies focused on the preparation methods^14–16^, physicochemical properties^17^, and formation mechanism of the materials^18,19^, and most of them used the same materials to prepare multilayer hydrogels^19–21^. Hydrogels with different gel layers from different materials are rare in constructing a drug delivery system.

Usually, hydrogels can be fabricated by either natural polymers or fully synthetic polymers^22^. The natural polymers such as sodium alginate (SA) and carboxymethyl cellulose (CMC) are well known for their excellent non-toxicity, biocompatibility and biodegradability^23,24^, which are usually prepared by physical or chemical crosslinking with crosslinkers^1,25^. Both SA and CMC are polyanionic polysaccharides containing abundant carboxyl group in the polymer chain, and easily form the hydrogel by Ca^2+^ crosslinking^26^. However, these physical hydrogels such as SA-Ca^2+^ system always possessed the notable drawbacks of instability and rapid dissolution^27^. On the contrast, the hydrogels formed by synthetic polymers by polymerization crosslinking or physiochemical crosslinking possessing the advantages of excellent mechanical strength and physicochemical stability^28,29^. But the hydrogels fabricated either by natural polymers or synthetic polymers with single network and microporous structure are always limited by the drug leakage problem that the drug concentrations can not be maintained effectively at the sustainable and desired levels for a longer period of time^30,31^. Therefore, in order to solve the problem caused by the microporous hydrogels structure, intelligent multi-component hybrid hydrogels with multilayer structure has become a potentially effective strategy^32,34^.

In our previous investigation, various strategies for regulating the sustained and controlled release speed of drugs were explored in order to solve the leakage and burst phenomenon caused by the pore size of gel network. A variety of structural type of hydrogel delivery systems was constructed, as shown in Fig. 1. It involves single network^35^, semi-interpenetrating networks (Semi-IPN)^36^, interpenetrating networks (IPN)^37^, membrane controlled IPN^38^, and nanoparticle doped composite networks^39^. It is worth to mention that the construction of membrane-controlled IPN hydrogel^37^ (as shown in Fig. 2) by polyelectrolyte complex coacervation on the outer surface of gel, greatly reduced the burst release phenomenon during drug delivery, especially in gastric acid environment.

**Figure 1:**
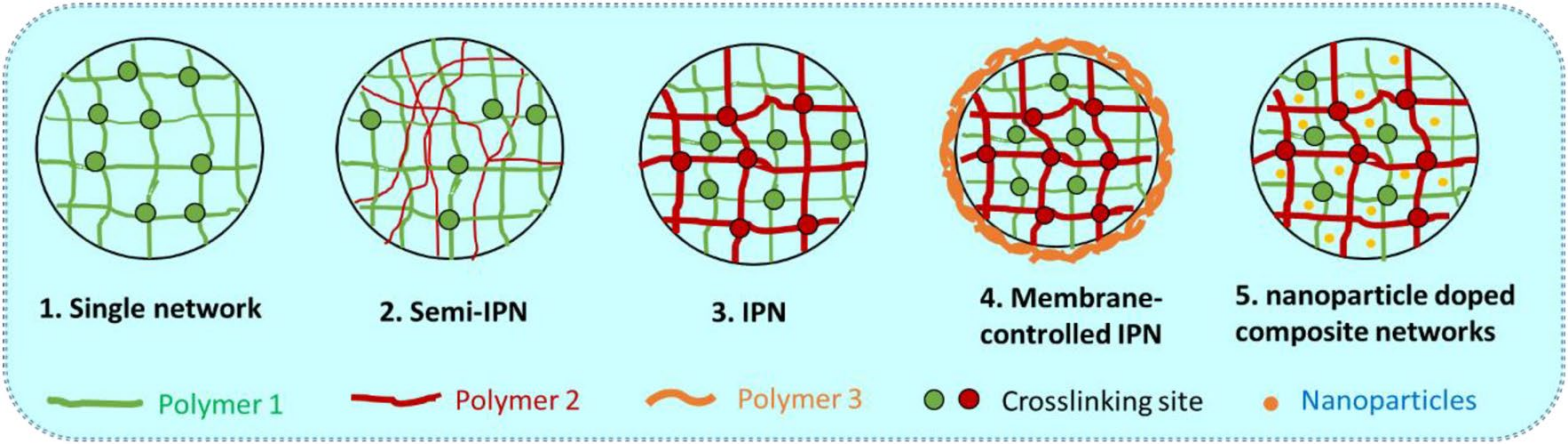
The illustration of five different network structure of hydrogels.

**Figure 2:**
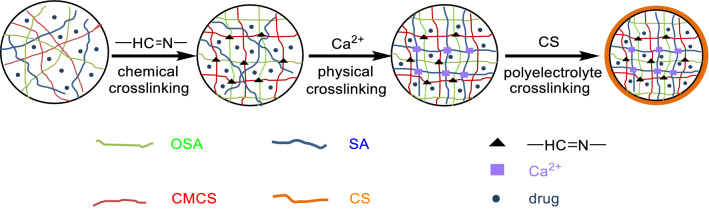
Preparation route of membrane-controlled interpenetrating network (IPN) hydrogel.

In this study, a double-layer hydrogel with different inner and outer structure was fabricated, in which the outer layer hydrogel formed by chemical crosslinking replace the membrane structure of previously prepared by polyelectrolyte coacervation (in Fig. 2). The double-layer hydrogels with polysaccharides inner core and synthetic polymer out-layer combining the advantages of two different hydrogel networks was produced. Natural polymers SA and CMC were used to form the inner layer with IPN structure by crosslinking with Ca^2+^ individually. Synthetic materials of poly(*N*,*N*-dimethylacrylamide) (PDMA) or poly (acrylamide) (PAA), which were polymerized by *N*,*N*-dimethylacrylamide (DMAA) and acrylamide (AA) monomer respectively, were widely used in the biomedical fields^40–42^. They were individually introduced to form the outer layer coating on the surface of SA-CMC hydrogel in order to enhance the strength and stability. The inner SA-CMC hydrogels, formed by ionic gelation effect, are pH-sensitive in the weak-alkaline intestinal environment to avoid the drug leakage problem in stomach. And the out layer hydrogels, fabricated by chemical crosslinking^19^, are physicochemically stable with little swelling behavior, which can further prevent the inner hydrogels expansion and the inner contents diffusion, finally realizing the drug sustainable release. The double-layer hydrogels were prepared by a simple and mild method, as depicted in Fig. 3. Different characterization methods were used to study the physical and chemical properties of the system. Moreover, by using hydrophilic small-molecule drugs of metformin (MH), hydrophobic small-molecule drugs of indomethacin (IDM), and large-molecule proteins of bovine serum albumin (BSA) as model drugs, the sustained and controlled-release behavior of the system in simulated gastric-intestinal fluid were investigated.

**Figure 3:**
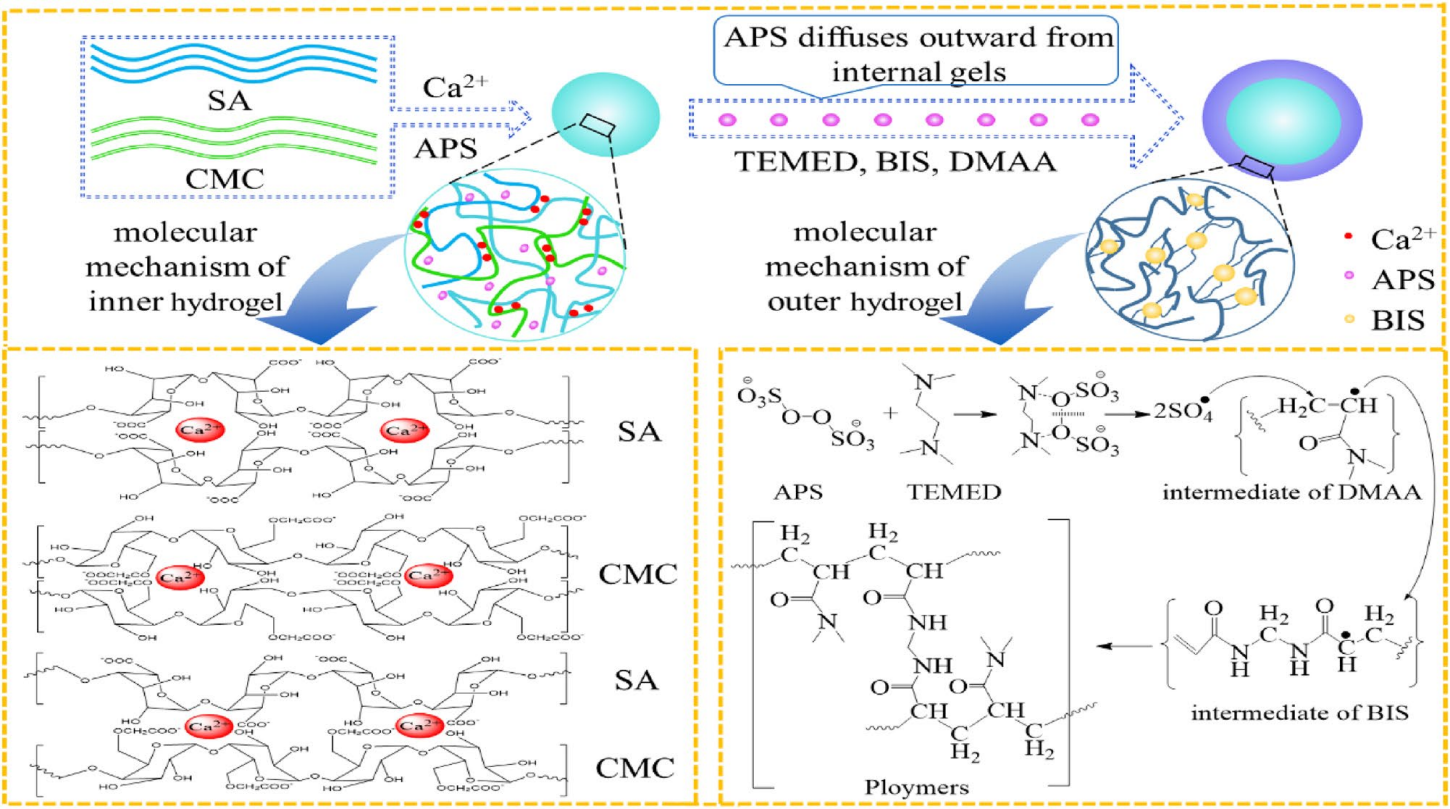
Formation mechanism of the double-layer hydrogels.

## Results and discussion

### Formation mechanism

In this study, double-layer hydrogels were prepared by the diffusion method (Fig. 3). The inner layer of the double-layer hydrogels was made of SA and CMC, which was dripped into the CaCl_2_ solution (with 1.5% APS). The formation mechanism was physical crosslinking between Ca^2+^ and COO^−^ and the inner diameter of the hydrogel can be controlled by modifying the composition ratio of SA to CMC^43^. The outer gel was formed by free radical polymerization of DMAA and AA monomer in the solution initiated by APS diffused from the inner hydrogel layer, and catalyzed crosslinking by BIS^44^. The synthetic polymer products of PAA and PDMA are nontoxic and widely used in drug delivery^45,46^. The function of the outer gel was regulated by optimizing the types and polymerization time of the monomers. The inner SA-CMC hydrogels based on two polyanionic polysaccharides are pH-sensitive in the weak-alkaline intestinal environment to avoid the drug leakage and burst release problem in stomach. And the out layer hydrogels, with well physicochemical stability, are great barrier to limit the expansion of inner hydrogels as well as the diffusion of inner contents, finally realize the drug sustainable release effect. Moreover, a two-layer hydrogel system with different internal/external structures and properties was formed through the selection and optimization of materials and crosslinking methods, which laid the possibilities for construction a novel oral sustained-release drug delivery system.

### Morphological studies

**Figure 4:**
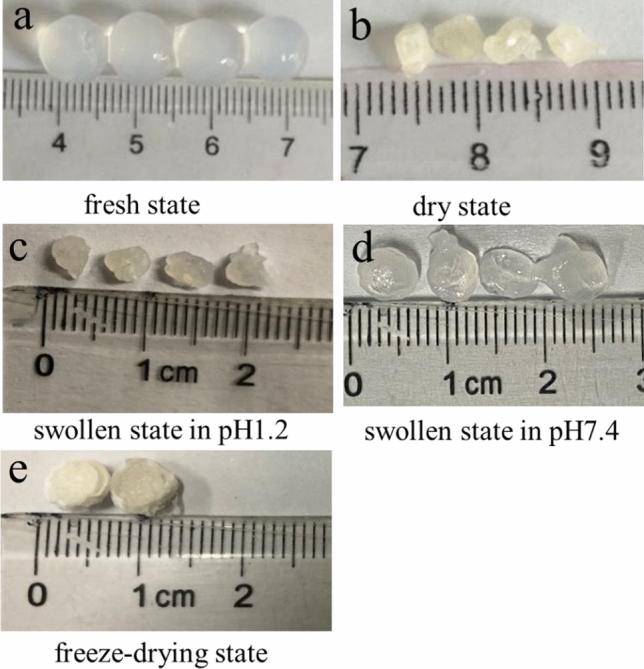
The morphologies of SA-CMC@PDMA-1 hydrogels in different state. [(**a**) in fresh state; (**b**) in dried state; (**c**) in swollen state at pH1.2; (**d**) in swollen state at pH 7.4; (**e**) in freeze-drying state].

Figure 4a shows the image of the newly prepared SA-CMC@PDMA-1 hydrogel, which had a diameter of approximately 7 mm. Figure 4b shows the hydrogels dried under natural conditions. The hydrogels were in an irregular shape after losing its moisture, and its diameter was reduced to approximately 4 mm. Figure 4c shows hydrogels at the swelling state after treatment in pH 1.2 buffer for 4 h. The out layer was swelling, whereas the inner layer was in shrunken state. Figure 4d shows hydrogels at the swelling state after being transferred from pH 1.2 into pH 7.4 buffer solution for 4 h. The outer layer was not swelling any longer, whereas the inner hydrogel gradually expanded under alkaline conditions. Therefore, the two layers of the hydrogels exhibited different properties: the inner layer was alkaline-sensitive, whereas the outer layer was without pH-sensitivity.

**Figure 5:**
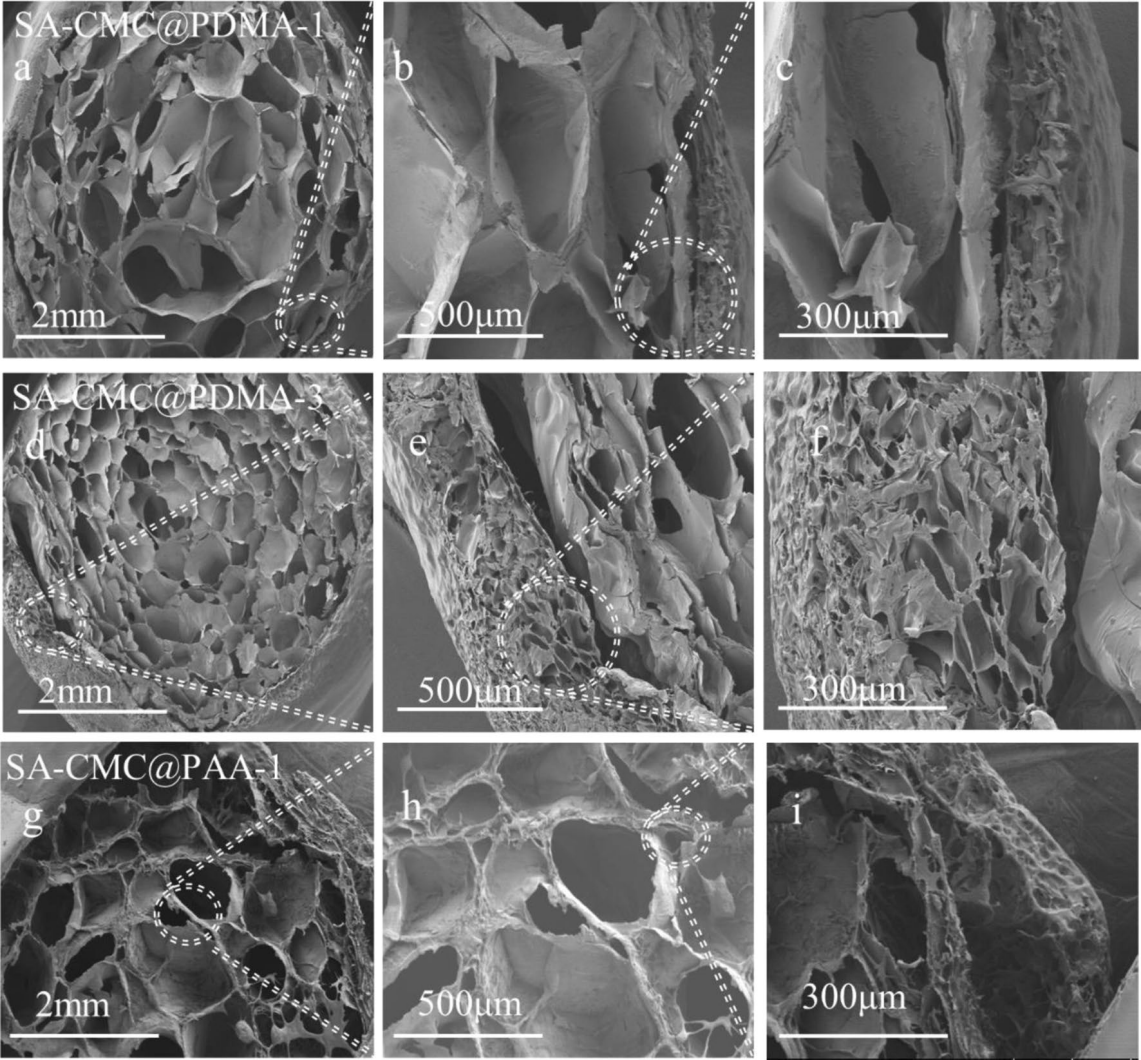
SEM images of double layer hydrogels with different magnification [(**a**–**c**) SA-CMC@PDMA-1; (**d**–**f**) SA-CMC@PDMA-3; (**g**–**i**) SA-CMC@PAA-1].

Figures 4e and 5 shows the surface and inner morphology of the hydrogels in freeze-drying state, respectively. After freeze-dried, the surface morphology of the hydrogel shrinks and shows a sponge state. As observed by SEM at different magnifications, Fig. 5a–c show SEM images of SA-CMC@PDMA-1 hydrogels with the inner layer (2.0% SA/0.5% CMC) and outer PDMA layers crosslinked for 1 min. Figure 5e–g show images of SA-CMC@PDMA-3 hydrogels with the inner layer (1.0% SA/1.5% CMC) and outer PDMA layers crosslinked for 2 min. All the inner and outer layers of the composite hydrogels showed a three-dimensional pore structure. The double-layer structure of the composite hydrogels was clearly observed in the images, and the boundary between the two layers was obvious. In addition, the two layers of the hydrogels showed different three-dimensional apertures owing to the different materials, and the pore size of the outer hydrogel was obviously smaller than that of the inner hydrogel. Figure 5c,g show that the thickness of the outer hydrogel increased from approximately 100–450 µm when the formation time of the outer layer was increased from 1 to 2 min. Figure 5a,e show that the three-dimensional aperture size was significantly altered with different proportions of SA and CMC. In summary, the unique double-layer structure of the hydrogels enabled the inner layer to facilitate drug loading, whereas the outer layer with compact structure effectively controlled drug release and achieved sustained drug release.

### FTIR characterization

**Figure 6:**
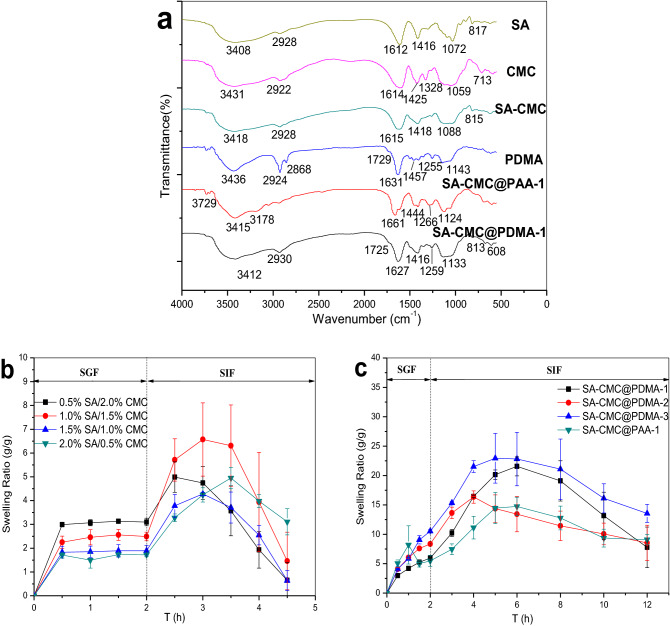
FTIR spectrum of SA, CMC, SA-CMC hydrogel, PDMA hydrogel, SA-CMC@PAA-1 and SA-CMC@PDMA-1 double layer hydrogel (**a**). Swelling profiles of hydrogels in simulated gastrointestinal fluid: (**b**) Single layer hydrogel with different inner layer composition; (**c**) SA-CMC@PDMA-1, 2, 3 hydrogel and SA-CMC@PAA-1).

Figure 6a presents the FTIR spectra of SA, CMC, SA-CMC hydrogels, PDMA hydrogels, SA-CMC@PAA-1 and SA-CMC@PDMA-1 double-layer hydrogels. All the FTIR spectra exhibited broad peaks around 3000–4000 cm^−1^, corresponding to the O–H stretching vibration modes. In the IR spectra of SA, the broad absorption peak at 1612 cm^−1^ corresponded to C=O asymmetric stretching vibration, and the narrow absorption peak at 1416 cm^−1^ corresponded to –COO^−^ symmetric stretching vibration^47,48^. In the IR spectra of CMC, the peak at 2922 cm^−1^ corresponded to –C–H stretching vibration, the peak at 1614 cm^−1^ corresponded to C=O stretching vibration, and the peak at 1059 cm^−1^ was due to C–O–C stretching vibration^48,49^.

In the IR spectra of SA-CMC hydrogels, the absorption peak at 2928 cm^−1^ was the stretching vibration absorption of C–H, the peak at 1615 cm^−1^ corresponded to the stretching vibration peak of C=O, the narrow peak at 1418 cm^−1^ corresponded to the symmetrical stretching vibration of –COO^−^, and the peak at 1088 cm^−1^ was due to C–O stretching vibration. In the spectrum of the composite hydrogels, there was no obvious new absorption peak from the inner layer samples. The main reason was that the three-dimensional network of the hydrogels was physically formed by Ca^2+^ crosslinking without new chemical bonds between the polymers.

The absorption peak of PDMA at the wavelength of 3436 cm^−1^ was caused by –NH stretching vibration, the absorption peak at 2924 cm^−1^ corresponded to –CH_2_– stretching vibration, and the absorption peak at 1631 cm^−1^ corresponded to the asymmetric stretching vibration of C=O in amide. One new absorption peak appeared at 1255 cm^−1^, which corresponded to the stretching vibration of amide^50^, implying that the polymer was formed by chemical crosslinking of DMAA. In SA-CMC@PAA-1 hydrogel, the absorption peak at 1661 cm^−1^ was corresponded to the asymmetric stretching vibration of C=O in amide by the polymerization of AA monomer.

### Swelling analysis

Figure 6b shows the swelling behavior of single-layer hydrogels with different inner structures. The four kinds of hydrogels did not swell in SGF, for the reason that many carboxyl groups existed in SA and CMC in the inner hydrogel matrix. In an acidic medium, –COOH did not ionize and had no electrostatic repulsion between the polymers. The hydrogel network structure was compact, the gap between polymers was small, and the hydrophilic property of SA and CMC decreased; thus, the swelling rate was low. However, with the decrease in CMC content, the swelling rate decreased. The reason was that the proportion of –COOH in the CMC structure was lower than that of SA, and the water absorption of SA was lower than that of CMC. In SIF, the swelling rate increased with time and then decreased. In an alkaline medium, –COOH was continuously dissociated and electrostatic repulsion force between –COO^−^ was formed and increased, thereby making the gel swelling and enlarging the network gap. The hydrophilic property of the network increased, and the gel absorbed a large number of water molecules, further increasing the swelling rate. In the later period, as the physical network formed by –COO^−^ and Ca^2+^ was unstable in SIF, the hydrogels gradually dissolved. Moreover, lower –COOH content led to weak crosslinking reaction with Ca^2+^ and led to faster disintegration rate of hydrogels. In conclusion, as the main limitation of SA gel reported previously, the research showed the similar results and the drug leakage frequently occurred in these kinds of single-layer hydrogels due to the macroporous structure of the gel network^37^. Hence, in order to solve the problem of the single-layer hydrogel, the double-layer hydrogel was fabricated in this research.

The matrix composition of the double-layer hydrogels in Fig. 6c was shown in Table 1. As shown in Fig. 6c, the swelling time of the double-layer hydrogel was extended significantly from 4.5 to 12 h through additional formulation of the outer gel. Although the two layers with distinct properties exhibited different swelling behavior in the later stage of swelling: the swelling of out layer tended to reach the balance, whereas the inner hydrogel layer disintegrated and eroded gradually due to the weak physical crosslinking (The appearance is shown in Fig. 4d), the total swelling ratio increased largely than the single layer hydrogel. After swelling in the SIF for 12 h, the out layer of the hydrogels still kept integrity and undegradation, providing better sustained-release effect for the drug delivery system. Comparison of curves SA-CMC@PDMA-2 and SA-CMC@PDMA-3 revealed that increasing crosslinking time of the outer layer hydrogel retarded the disintegration time of the inner layer hydrogel due to the increasing thickness of out-layer hydrogel, which would be benefit to the sustained and controlled drug-release effect of the double-layer hydrogel. Comparison of curves SA-CMC@PDMA-1 with SA-CMC@PDMA-2 showed that decreasing SA content decreased the swelling property of the composite hydrogels, thus realized the sustainable drug release in the SIF environment. The SA-CMC@PDMA hydrogels with PMDA as outer-layer exhibited better swelling ability than SA-CMC@PAA-1.

**Table 1:** The composition of double-layer hydrogels.

Formulation name	Inner layer composition (SA/CMC, w/w)	Outer-layer material	Outer-layer crosslinking time (min)
SA-CMC@PDMA-1	2.0%/0.5%	DMAA	1
SA-CMC@PDMA-2	1.0%/1.5%	DMAA	1
SA-CMC@PDMA-3	1.0%/1.5%	DMAA	2
SA-CMC@PAA-1	2.0%/0.5%	AA	1

### Thermogravimetric analysis

**Figure 7:**
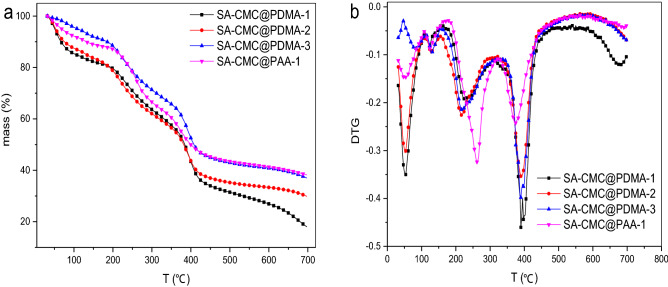
(**a**) The TG curves of SA-CMC@PDMA-1, 2,3 and SA-CMC@PAA-1; (**b**) The DTG curves of SA-CMC@PDMA-1, 2,3 and SA-CMC@PAA-1.

Thermogravimetric analyzer was used to analyze TG and DTG profile of double layer hydrogels (Fig. 7). Largely, there are three major weight losses in hydrogels: the first one below 100 °C is due to water desorption, the second one at 270 °C is due to destruction of glycosidic bonds, and the third one at about 400 °C is due to destruction of out layer polymer. Incorporation of out layer polymer into the composite hydrogels brought about certain thermal stability and some notable changes in the degradation pattern. In the third phase, the degradation degree was prominently delayed primarily due to the synthetic polymer in the outer layer of the hydrogels. Thus, the out layer prepared by PDMA or PAA polymer slightly improved the thermal stability of the composite hyrdogels at the degradation phase.

### Rheology analysis

**Figure 8:**
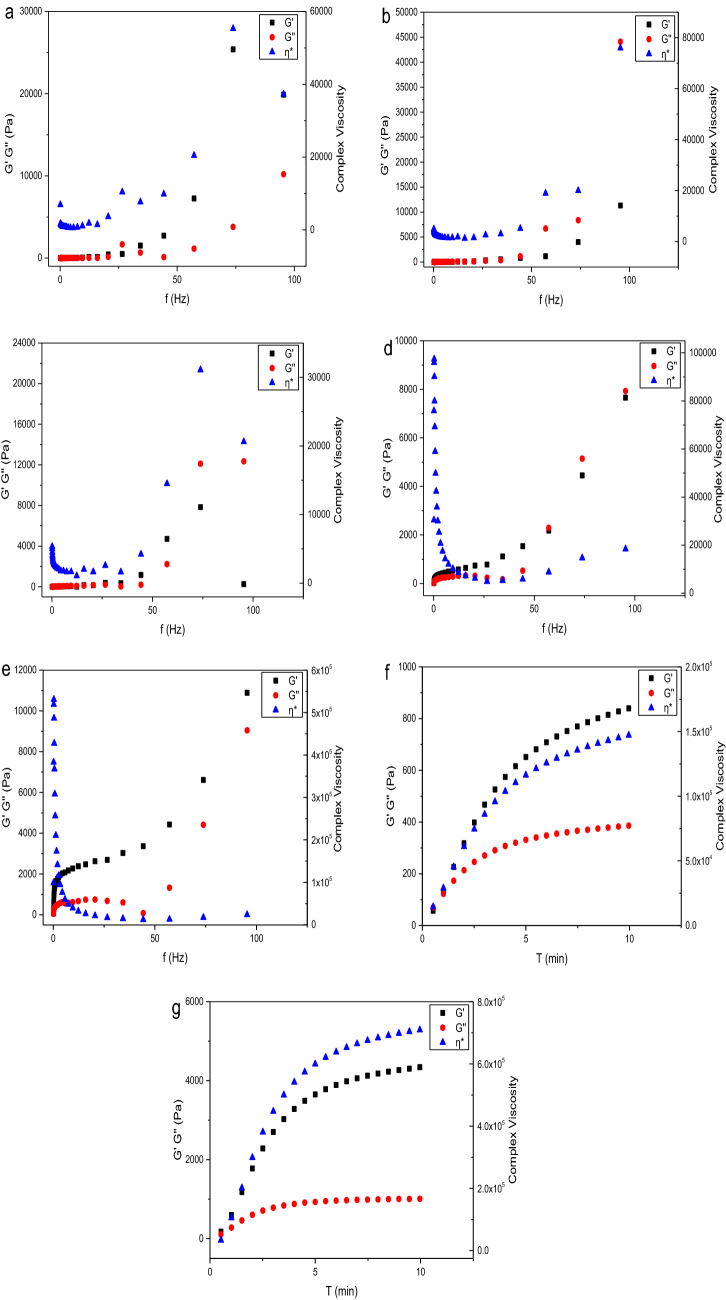
Rheological test [(**a**–**c**) the frequency sweep test of SA-CMC, PDMA, PAA solution; (**d**,**e**) the frequency sweep test of the crosslinking process of SA-CMC@PDMA-1and SA-CMC@PAA-1, respectively; (**f**,**g**) time test of the crosslinking process of SA-CMC@PDMA-1and SA-CMC@PAA-1].

It can be seen from the Fig. 8 that the G′ and G″ of the SA-CMC, PDMA or PAA solution do not change significantly within the test frequency range, but when the two solutions of inner layer materials and out layer materials are mixed, it can be seen that G′ and G″ increase significantly, which indicates that the crosslinking reaction occurs between the two solutions. In addition, in the time test, after the polymer solution is mixed, G′ and G″ first increase sharply, and then change slowly, which can also explain the crosslinking reaction between the two solutions. The gel images and rheological data indicated that the outer polymer PDMA or PAA can form a stable layer out of the SA-CMC during gel preparation.

### Effect of outer layer thickness on drug release behavior

**Figure 9:**
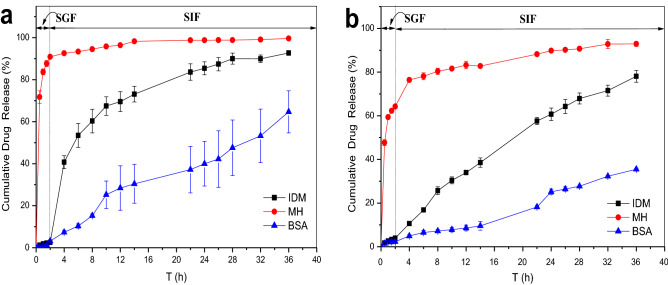
Effect of outer layer thickness on the cumulative drug release behavior of double layer hydrogels in simulated gastrointestinal fluid [(**a**) SA-CMC@PDMA-2, external layer formation time: 1 min; (**b**) SA-CMC@PDMA-3, external layer formation time: 2 min].

Cumulative release profiles of the bilayer hydrogels with different outer layers in simulated gastrointestinal fluid are shown in Fig. 9. As shown in Fig. 9a, the double-layer hydrogels had good sustained-release effect for IDM and BSA. Within the first 2 h, the outer hydrogel in SGF expanded and swelled, the inner hydrogel, which had a pH-sensitive structure, did not swell or resolve; thus, the drug was not released at all. In SIF, the inner hydrogel began to swell and the network structure opened, thereby releasing IDM and BSA gradually. The IDM release rate reached more than 90% at 36 h and the BSA release rate reached 64%. The hydrogels showed no controlled-release effect for MH, and the MH release rate at 2 h reached approximately 90% owing to the severe burst-release effect.

As shown in Fig. 9b, the double-layer hydrogels had good sustained-release effect in IDM and BSA. The outer layer hydrogel in SGF swelled within the first 2 h, but the drug was not released. In SIF, the inner hydrogel began to swell, the network structure opened, and the IDM was released slowly. At 36 h, the release rate reached approximately 80% for IDM. On the contrary, the hydrogel exerted a burst-release effect for MH in SGF, and the drug release at 2 h reached approximately 65%, whereas BSA was not released in SGF. There are two main reasons for this result. MH is a water-soluble small molecule, and is easy to diffuse through the hydrogel barrier to the aqueous solution. Therefore, MH has been spread to the outer layer in the preparation process, and the drug will still diffuse into the SGF from the non-swelling inner hydrogel. The synergistic effect of these two causes led to the sudden release of MH in gastric acid. However, the molecular size of BSA is large enough to be detained by the pore size of the bilayer hydrogel. As a result, after being transferred to SIF, the release rate of BSA was still relatively slow, and the release rate was only approximately 35% after 36 h.

The cumulative drug-release curves in Fig. 9a,b show that the release rate of drugs in the inner hydrogel was significantly retarded owing to diffusion resistance by increasing the thickness of the outer layer. Based on the physicochemical properties of different drugs, the formation time of the outer layer of the double-layer hydrogels can be optimized to achieve the best-sustained-release effect.

### Effect of inner matrix composition on drug release behavior

**Figure 10:**
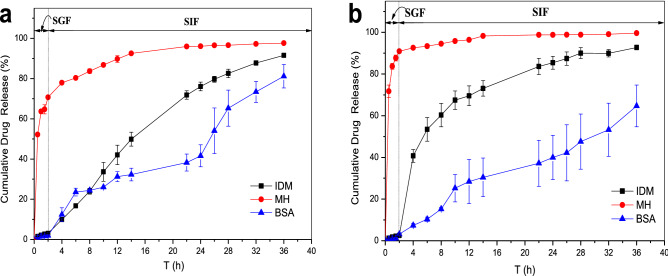
Effect of inner matrix composition on the cumulative drug release behavior of double layer hydrogels in simulated gastrointestinal fluid [(**a**) SA-CMC@PDMA-1; (**b**) SA-CMC@PDMA-2].

The cumulative release behavior of the bilayer hydrogels with different inner material compositions in simulated gastrointestinal fluid is shown in Fig. 10. The hydrogels exhibited good sustained-release effect and pH-dependent release profile for IDM and BSA, with IDM release rate of more than 90% at 36 h and BSA release rate of 81%. However, the hydrogels exerted no sustained-release effect for MH, and owing to the burst-release effect, the drug release at 2 h reached approximately 70%.

Figure 10a,b indicated that the changes in the material proportions of the inner hydrogel significantly affected the release of BSA, as the composition of the inner layer was directly related to the pore size of the network. According to the SEM images of the single inner hydrogel, the pore size of the 1.0% SA/1.5% CMC hydrogels was obviously smaller than that of the 2.0% SA/0.5% CMC hydrogels, therefore, drug release from SA-CMC@PDMA-2 hydrogels was slower than that from SA-CMC@PDMA-1 hydrogels. However, the effect of the double-layer hydrogels on the release of MH and IDM was not significant. The main reason was that comparing with the protein drugs, the pore size of the gel inner network was much larger than that of small-molecule drugs; thus, the change in network pore size had little effect on small-molecule drugs.

### Effect of material type of the outer layer on drug release behavior

**Figure 11:**
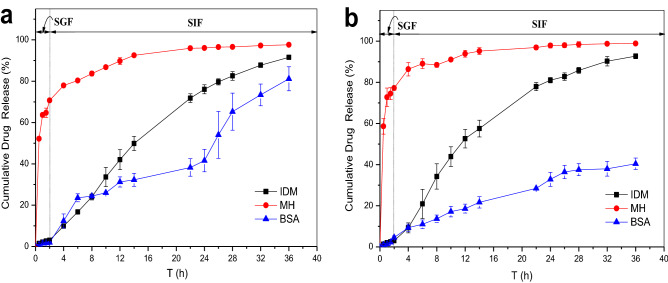
Effect of outer-layer materials type on the cumulative drug release behavior of double layer hydrogels in SBF [(**a**) SA-CMC@PDMA-1; (**b**) SA-CMC@PAA-1].

The cumulative release behavior of the double-layer hydrogels with different outer materials in simulated gastrointestinal fluid is shown in Fig. 11. The release effect of the hydrogels with PAA outer layer on IDM and MH was not significantly different from that of the hydrogels with DMAA outer layer under the same preparation conditions, but the cumulative release rate of BSA decreased significantly. This result indicated that the hydrogels with the outer-layer polymer composed of PAA was more effective than that of hydrogels with PMAA outer layer in realizing sustained-release of BSA due to the weak swelling behavior and small pore size in the PAA.

### Stability of the released BSA

**Figure 12:**
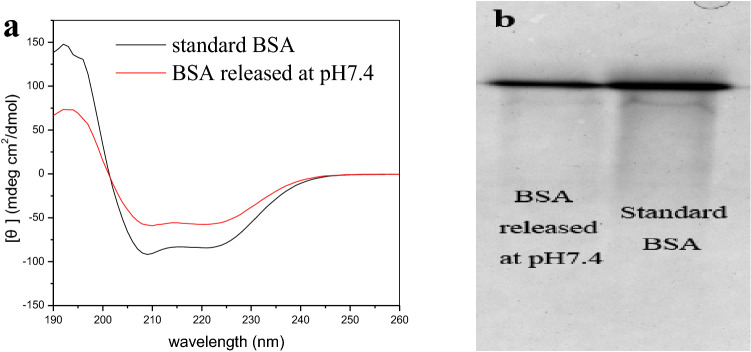
Stability determination of the released BSA [(**a**) CD spectra of standard BSA and BSA released from the SA-CMC@PDMA-3 in SIF; (**b**) SDS-PAGE gel of the BSA released from the SA-CMC@PDMA-3 in SIF and BSA standard].

To study the stability of BSA released from the hydrogels, circular dichroism (CD) spectroscopy and electrophoretic analysis were conducted and the results are shown in Fig. 12. The spectra of BSA released from SIF showed characteristics band of alpha helical structure at 208–222 nm^51^, similar to the standard BSA in Fig. 12a, there was no notable conformation change for the BSA released from the double-layer hydrogels in simulated gastrointestinal medium  (as shown in the Supplementary information). The result suggests that the BSA conformation was largely preserved during loading and delivery process.

Figure 12b indicated that the protein bands of the experimental samples were consistent with the bands of the standard products, indicating that the BSA in the hydrogels remained stable in the preparation and release environment, and that the preparation environment of the hydrogel did not destroy its structure.

## Conclusions

In this study, double-layer hydrogels with polysaccharides inner core and synthetic polymer out-layer combining the functions of two different hydrogel networks was produced. The core matrix of the inner hydrogel was based on SA and CMC and was formed by physical crosslinking of Ca^2+^ under mild and easy operation conditions. Acrylamide and its derivatives were used for producing the outer layer of the hydrogels by free radical polymerization, allowing fast chemical crosslinking speed, controllable crosslinking conditions, and good stability.

The double-layer structure and three-dimensional crosslinking network of the bilayer hydrogels were characterized. By studying the swelling behavior of the hydrogels, the ratio of the materials of the inner matrix in the double-layer hydrogels was optimized. Furthermore, the swelling time of the double-layer hydrogel could be extended from 4.5 to 12 h through additional formulation of the outer gel, thus allowing extended drug release. The cumulative drug-release behavior of three different drug types indicated that changes in the material composition and outer-layer material types of the macroporous hydrogels led to significant sustained release of macromolecular drugs, but had little effect on small-molecule drugs. Change in the thickness of the outer layer of the hydrogels had obvious effect on the release of the three model drugs. The increase in the outer layer thickness was beneficial to increase drug diffusion resistance and delay drug release.

In summary, the double-layer hydrogel system showed better sustained-release effect and less drug leakage phenomenon than that of monolayer hydrogels, especially for the macromolecule drugs and hydrophobic drugs. In order to complement the hydrogel properties, different composite materials can be used to fabricate the distinctive gel layer. In addition, the multi drug delivery and sequential drug release can be realized in the double layer structure, and the drugs can be loaded into different gel layers to achieve the independence of multiple drugs in the same vehicle. Moreover, sequential drug release was optimal to adapt to the complex physiological environment of human body and achieve more accurate treatment. Therefore, the double-layer hydrogels have great potential in developing into a novel functional sustained drug delivery system.

## Experimental

### Chemicals and materials

Sodium alginate (SA, M_w_: 4.2 × 10^5^, M/G = 1.52), carboxymethyl cellulose (CMC, Viscosity: 1000–1400 mpa s, SD = 1.2), methyl cellulose (MC, Viscosity: 1500mpa.s), acrylamide (AA), *N*,*N*-dimethylacrylamide (DMAA), *N*,*N*-methylenedibis-propionamide (BIS), tetra-methylethylenediamine (TEMED), metformin (MH) and indomethacin (IDM) were purchased from Aladdin Reagent Co., Ltd. (Shanghai, China). Phosphoric acid was obtained from Xinyang Chemical Co., Ltd. (Henan, China). Bovine serum albumin (BSA) and Coomassie brilliant blue were purchased from Sigma Chemical Company (St. Louis, MO, USA). Ammonium persulfate (APS), potassium dihydrogen phosphate, sodium hydroxide, concentrated hydrochloric acid, and calcium chloride were purchased from Sinopharm Chemical Reagent Co., Ltd. (Shanghai, China).

### Preparation of inner hydrogel

**Figure 13:**
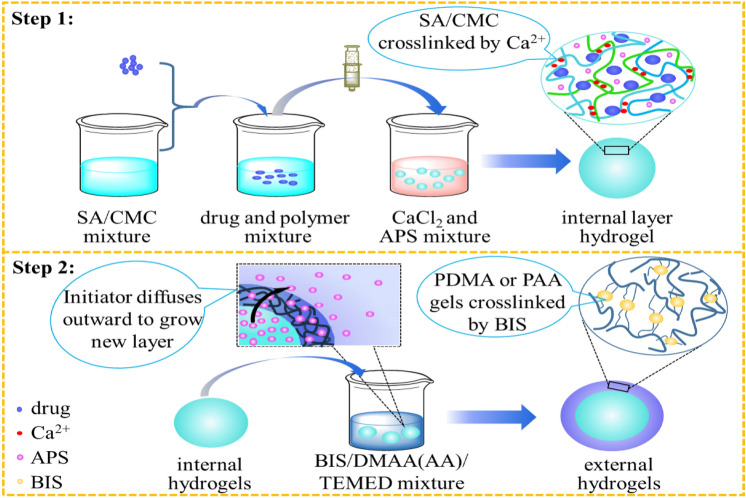
Preparation schematic diagram of double-layer hydrogel.

A mixed solution of SA/CMC with different mass ratio (2.0%/0.5%, 1.0%/1.5%, 1.5%/1.0%, 0.5%/2.0%; w/w) was prepared. Then, the mixture was drop to 5% (w/w) CaCl_2_ solution dissolved by 1.5% ammonium persulfate (APS, w/w). Then, the mixed solution was stirred and crosslinked for 20 min to form an inner layer hydrogel core for next step. The two formulations of the inner layer hydrogel with the largest swelling rate and the longest swelling time were selected for the preparation of the double layer hydrogel. The preparation routine of the inner hydrogels was shown in Fig. 13.

To prepare drug-loaded double-layer hydrogels, drug was added to the SA/CMC mixture at a final concentration of 10 mg/mL. The subsequent preparation procedure of drug-loaded double-layer hydrogels are similar to the aforementioned preparation process.

### Preparation of double-layer hydrogel

In order to prepare multiple types of double-layer hydrogels (Table 1), the inner layer hydrogels were prepared by different polymer proportions and the external layer hydrogels (marked as PDMA and PAA) were employed by using 2 different synthetic materials (DMAA and AA). A certain amount of MC (3%, w/w) was weighed and swelled in the water. DMAA or AA (1 mol/L), BIS (2.2% mol/L) and TEMED (15 mg/mL) were added. In the mixture, MC acts as a suspending agent in the preparation of the external hydrogel. Then, the newly prepared inner hydrogels were transferred to the DMAA or AA mixture, and soaked for different times until the double-layer hydrogel with different outer-layer thickness was obtained. The unreacted monomers were removed by stirring and washing with purified water for 15 min. The preparation routine of the external-layer hydrogel was shown in Fig. 13.

### Morphological characterizations

The external morphology of SA-CMC@PDMA-1 was recorded by digital camera under the conditions of fresh preparation state, nature-drying state and swelling state at different pHs.

Scanning electron microscopy was performed on the double layer hydrogels (freeze-dried to maintain the porous structure without any collapse) to obtain information on the pore structure of hydrogels. The sufficiently swollen hydrogels were removed from the solution, quickly frozen in liquid nitrogen, and then freeze-dried under vacuum at room temperature for 3 days to remove the imbibed water completely until the samples became completely dry prior to morphological observation. The freeze-dried hydrogels were carefully cut to two halves to reveal the interior and mounted onto the base plate and coated with gold. The interior morphology of the swollen hydrogels at room temperature were observed using a scanning electron microscope (SEM, JEOL JSM-6700F, Japan) at different magnifications.

### FTIR spectroscopic analysis

The chemical structure of SA, CMC, SA-CMC, DMAA, and gels was characterized by infrared (IR) spectroscopy. The sample was mixed with KBr powder at a ratio of 1:100 and pressed into tablets. The spectra were measured on a Fourier transform (FT)-IR spectrometer (Spectrum One; PerkinElmer Inc., Waltham, MA, USA) in the wavelength range of 400–4000 cm^−1^ with a resolution of 4 cm^−1^.

### Swelling characteristics

The inner hydrogels and the double-layer hydrogels were naturally air-dried and weighed an appropriate amount of sample. The swelling-behavior experiment was performed in simulated gastric fluid (SGF, pH = 1.2) for 2 h, and followed by simulated intestinal fluid (SIF, pH = 7.4) until the weight reach to balance or lose. At time intervals of 30 min, the samples were separated from the medium. Wiped gently with filter paper, weighed precisely and recorded immediately. The swelling rate was calculated using the following formula^52^:

1 $${\text{SR }} = \, \left[ {\left( {{\text{m}}_{0} - {\text{m}}_{{\text{t}}} } \right) \, /{\text{m}}_{0} } \right],$$

where SR is the swelling ratio of the hydrogel, m_t_ (g) is the mass of the gel in swollen state at time t, and m_0_ is the initial mass of the hydrogel.

### Thermogravimetric analysis

Thermogravimetric analysis (TGA) of the dry hydrogels samples (5 mg) was determined on a TGA (TG 209F3, NETZSCH, Germany) at a heating rate of 10 °C min^−1^ under N_2_ atmosphere from 40 to 500 °C (Supplementary Information).

### Rheology analysis

After the inner polymer solution and the out layer monomer solution is mixed uniformly, the solution is dropped onto the plate of HAAKE rheostress 6000 (Thermo Scientific, USA) with a rubber tip dropper. The diameter of the plate is 35 mm, the gap is set at 1 mm, and the temperature is 25 ± 0.1 °C. In the frequency sweep test, the elastic modulus (G′), viscosity modulus (G″) and apparent viscosity (η*) were measured in the frequency range of 0.1–100 Hz with a frequency of 1.0 Hz and a strain rate of 1.0%. In the time test, the G′, G″ and η* were measured at a frequency of 1.0 Hz and a strain rate of 1.0%^53,54^.

### In vitro release behavior

To study the drug release profile in simulated gastrointestinal conditions, three drugs, BSA, MH, and IDM, with different molecular weights and solubility properties were chosen as the model drugs. The drug-loaded gels were immersed in SGF for 2 h and then transferred into SIF under 37 °C. Each weighed hydrogels was immersed in 50 mL of dissolution medium shaken in a rotary water bath shaker with 100 rpm at 37 °C. At an appropriate interval, a certain amount of dissolution solution was withdrawn and immediately replaced with an equal volume of fresh buffer in order to maintain the sink condition. Using a UV spectrophotometer (UV–Vis Spectrophotometer; Lambda 35, Perkin-Elmer, Norwalk, CT, USA), the amount of Metformin hydrochloride (MH) or indomethacin (IDM) were determined at 233 or 320 nm, respectively, and calculated with a standard curve equation. The BSA concentration was measured using a Bradford protein assay at λ = 595 nm. The cumulative release rate of the hydrogels^38,55^ was calculated as follows:

2 $${\text{Q}}_{{\text{n}}} (\% ) = \frac{{V_{0} C_{n} + VC_{{{\text{n}} - 1}} }}{{\text{W}}} \times 100,$$

where, C_n_ and C_(n−1)_ are the drug concentration for sampling for n times and n − 1 times, V_0_ is the initial volume of the drug release medium, V is the sampling volume, and W is the drug mass loaded in the gels.

### BSA stability study

The stability and conformation of the released BSA from the double-layer hydrogels was determined by circular two color spectrophotometry (CD, Jasco J-810 spectropolarimeter, Japan)^56^. The BSA concentration released from the hydrogel was diluted to the appropriate concentration before analysis. CD analysis was performed using a quartz cell with 0.1 cm path length and the following parameters: 1 nm bandwidth, 10 mdeg sensitivity, 0.2 nm resolution, 4 s response time and 100 nm min^−1^ scan. The samples were scanned in the ultraviolet range of 190–260 nm for structural analysis. The BSA structural integrity was determined by SDS–polyacrylamide gel electrophoresis (PAGE)^38,43^.

### Statistical analysis

All the experiments were performed at least three times and the results were presented as means ± standard deviation (SD). Statistical analysis was performed in Origin 8.0. In all the tests, the statistical significance was set at a value of p < 0.05.

## Supplementary Information

Supplementary Information.
