# Supplementary material for: A double-layer hydrogel based on alginate-carboxymethyl cellulose and synthetic polymer as sustained drug delivery system
Source: Sci Rep. 2021 Apr 28;11:9142. doi: 10.1038/s41598-021-88503-1 (PMC8080826; doi:10.1038/s41598-021-88503-1)
Supplement: Supplementary file 1 — Supplementary Information. [file 41598_2021_88503_MOESM1_ESM.pdf]

A double-layer hydrogel based on alginate-carboxymethyl cellulose and synthetic polymer as sustained drug delivery system

Yan Hu[1,2,*], Sheng Hu[1,2], Shangwen Zhang[1,2], Siyi Dong[1,2], Jie Hu[1,2], Li Kang[1,2,*], Xinzhou Yang[1,2]

(1. School of Pharmaceutical Science, South-Central University for Nationalities, Wuhan 430074, China; 2. National Demonstration Center for Experimental Ethnopharmacology Education, South-Central University for Nationalities, Wuhan 430074, China)

[*]Corresponding author Tel.: +8627-67841196; Fax.: +8627-67841196
E-mail address:huyan200277@163.com.
[*] Corresponding author Tel.: +8627-67841196; Fax.: +8627-67841196
E-mail address: Kang_11@126.com.

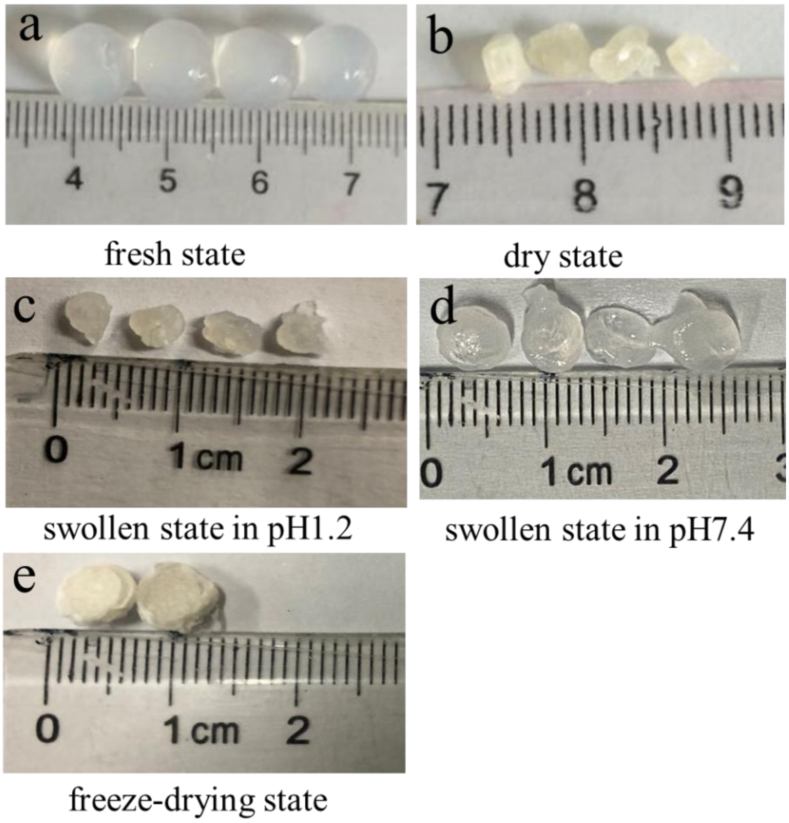

Figure S1. The morphologies of SA-CMC@PDMA-1 hydrogels in different state. (a. in fresh state; b. in dried state; c. in swollen state at pH1.2; d. in swollen state at pH 7.4; e. in freeze-drying state)

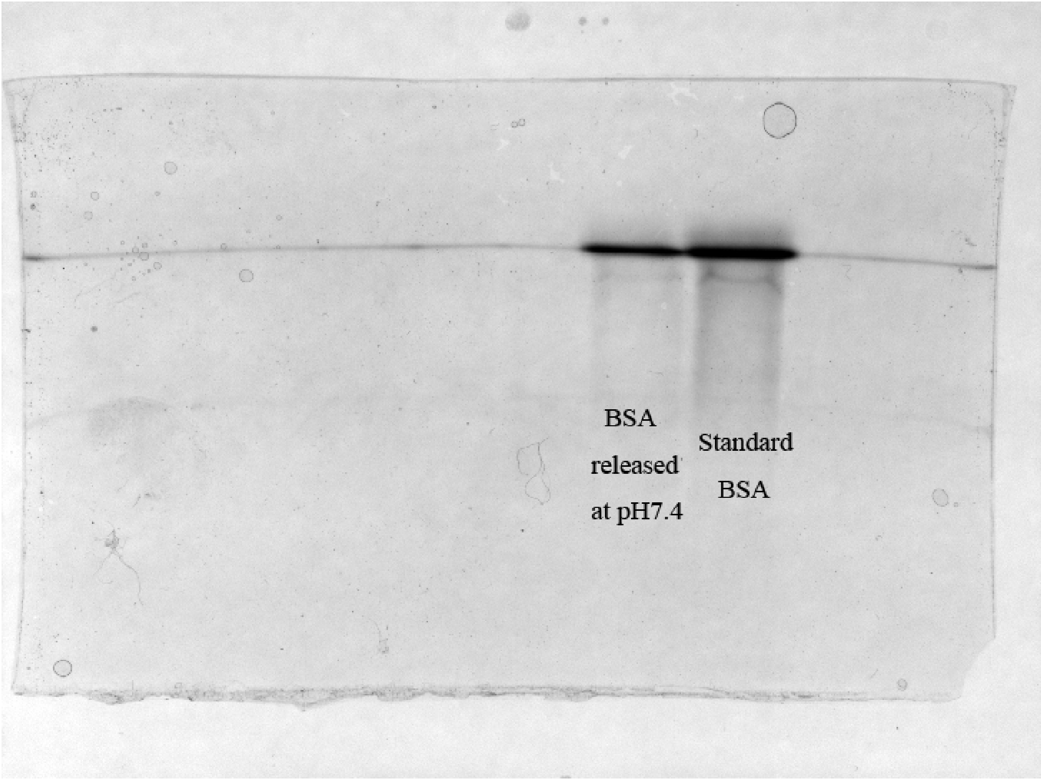

Figure S2 The SDS-PAGE gel of the BSA released from the SA-CMC@PDMA-3 in SIF and BSA standard. (The full image)
